# Relationship between abnormalities detected by magnetic resonance imaging and knee symptoms in early knee osteoarthritis

**DOI:** 10.1038/s41598-021-94382-3

**Published:** 2021-07-26

**Authors:** Seiya Ota, Eiji Sasaki, Shizuka Sasaki, Daisuke Chiba, Yuka Kimura, Yuji Yamamoto, Mika Kumagai, Masataka Ando, Eiichi Tsuda, Yasuyuki Ishibashi

**Affiliations:** 1 Department of Orthopaedic Surgery, Hirosaki University Graduate School of Medicine, Hirosaki, Aomori Japan; 2 Department of Active Life Promotion, Hirosaki University Graduate School of Medicine, Hirosaki, Aomori Japan; 3 Department of Diet and Health Sciences, Hirosaki University Graduate School of Medicine, Hirosaki, Aomori Japan; 4 Department of Rehabilitation Medicine, Hirosaki University Graduate School of Medicine, Hirosaki, Aomori Japan

**Keywords:** Epidemiology, Pain

## Abstract

We investigated the prevalence of magnetic resonance imaging (MRI) findings and their relationship with knee symptoms in women without radiographic evidence of knee osteoarthritis (KOA). This cross-sectional cohort study included 359 Japanese women without radiographic evidence of KOA (Kellgren‒Lawrence grade < 2). All participants underwent T2-weighted fat-suppressed MRI of their knees. Structural abnormalities (cartilage damage, bone marrow lesions [BMLs], subchondral cysts, bone attrition, osteophytes, meniscal lesions, and synovitis) were scored according to the whole-organ MRI score method. Knee symptoms were evaluated using the Knee Injury and Osteoarthritis Outcome Score. Participants were divided into early and non-KOA groups based on early KOA classification criteria. Logistic regression analysis was performed to evaluate the relationship between MRI abnormalities and knee symptoms. Cartilage damage was the most common abnormality (43.5%). The prevalences of cartilage damage, BMLs, subchondral cysts, bone attrition, meniscal lesions, and synovitis were higher in patients with early KOA than in those without. Synovitis (odds ratio [OR] 2.254, *P* = 0.002) and meniscal lesions (OR 1.479, *P* = 0.031) were positively associated with the presence of early KOA. Synovitis was most strongly associated with knee pain and might be a therapeutic target in patients with early KOA.

## Introduction

Knee osteoarthritis (KOA) is a major public health problem in the middle-aged to elderly population and negatively affects patients’ activities of daily living (ADL) and quality of life (QOL)^1–3^. Early diagnosis and treatment of KOA are required to reduce the health care burden^4^ by preventing disease development and progression^5–8^. Early KOA is characterized by the presence of knee symptoms in the absence of definitive radiographic abnormalities (Kellgren‒Lawrence [KL] grades 0‒1). Adults with KOA are more likely to report more knee symptoms the year before they develop radiographic evidence of KOA^9,10^. Based on this evidence, patients with early KOA may need to be targeted for therapeutic interventions as early as possible.

Pathologic changes found in osteoarthritic joints include degradation of the articular cartilage, thickening of the subchondral bone, osteophyte formation, varying degrees of synovial inflammation, degeneration of ligaments and menisci, and hypertrophy of the knee joint capsule^11^. Although radiographic evaluation cannot detect minute structural abnormalities, such as cartilage degeneration, meniscal lesions, synovitis, bone marrow lesions (BMLs), and subchondral cysts, magnetic resonance imaging (MRI) may allow the detection of these pathological joint tissue changes. In particular, along with knee symptoms, cartilage damage, meniscal lesions, and BMLs detected by MRI are included in the diagnostic criteria for early KOA^5,12^.

However, data on the structural abnormalities present in knees without radiographic changes and structural changes associated with knee symptoms are scarce. Therefore, the present study aimed to examine the cross-sectional association between MRI lesions and knee symptoms in middle-aged women without radiographic features of KOA. We hypothesized that the presence of cartilage damage, meniscal lesions, and BMLs are associated with knee symptoms, in accordance with the diagnostic criteria for early KOA.

## Methods

### Study design and participants

The participants had previously volunteered for the Iwaki Health Promotion Project, which is a community-based preventive medicine program that aims to improve the average life expectancy by conducting general health checkups and prophylactic interventions, as previously described^13–16^. The ethics committee of the Hirosaki University Graduate School of Medicine approved the study (reference number: 2017-026 and 2019-009), and all subjects provided written informed consent before participation. All methods were performed in accordance with the relevant guidelines and regulations.

**Figure 1:**
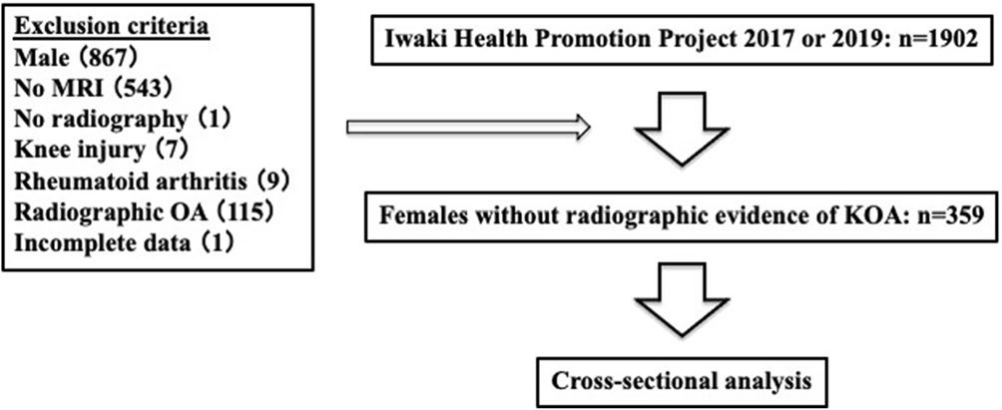
Flow of participants enrollment in Iwaki Health promotion project. Finally, 359 females were enrolled for the statistical analysis.

In total, 2138 volunteers (876 men and 1262 women) were enrolled in the project in 2017 and 2019. In this study, the analysis of those who participated in both 2017 and 2019 (n = 236) was performed using data from 2017. The participants answered questionnaires about their medical history and current health, lifestyle, and disease-specific information, such as knee symptoms. Height and weight were measured and recorded by the project team staff, and the body mass index (BMI) was calculated. In this study, we focused on women without radiographic abnormalities because the prevalence of KOA is higher in women than in men^1,17^. The exclusion criteria of this study were as follows: men (n = 867) and women who lacked knee magnetic resonance (MR) images (n = 543), did not undergo radiographic evaluation (n = 1), had a history of rheumatoid arthritis (n = 9) or knee injury (n = 7), had radiographic features of KOA in either knee (n = 115), and had incomplete data (n = 1). Finally, 359 women without radiographic abnormalities were included in the analysis (Fig. 1).

### Radiographic evaluation

The presence of KOA was evaluated using weight-bearing and anterior–posterior radiographs of both knees. The beam was positioned parallel to the floor, with no angle, and aimed at the joint space. KOA severity was classified according to the KL classification^18^. KOA was diagnosed as KL grade ≥ 2 in the most affected knee. All joints were graded by two orthopedic surgeons (DC and ES), and any discrepancies were resolved by mutual consultation. As aforementioned, only subjects with no radiographic evidence of KOA were included in this analysis. The intra-rater and inter-rater reliabilities, expressed as interclass correlation coefficients (ICC) (1,1) and (2,1) of radiographic evaluation, were 0.952 and 0.931, respectively.

### Knee symptoms

Knee symptoms were scored using a patient-based outcome score, the Knee Injury and Osteoarthritis Outcome Score (KOOS). All participants completed the KOOS questionnaire independently. The KOOS questionnaire is a 42-item, knee-specific, self-administered questionnaire with five subscales: pain, symptoms, ADL, sports and recreation (sports), and knee-related QOL. All items were scored from 0 to 4, and the scores were then summed. Next, the raw scores were transformed to a 0–100 scale, with 100 representing the best result and 0 representing the worst^19,20^. Participants were divided into early KOA and non-KOA groups on the basis of their KOOS: a knee was defined as symptomatic when the scores for two of the four KOOS subscales (except KOOS sports) were “positive” (below 85%), according to the established classification criteria for diagnosing early KOA^6^. Additionally, participants with joint-line tenderness or crepitation of the knee according to the symptomatic knee were classified as having early KOA. Furthermore, in this study, in order to determine the association between MRI findings and knee pain, a score from 1 to 4 in each KOOS Pain item was defined as “positive,” and a score of 0 was defined as “negative”.

### Knee magnetic resonance imaging

All participants underwent MRI of the right knee using a rapid extremity coil and mobile MR unit (1.5 T; ECHELON RX, Hitachi, Tokyo, Japan) within 1 week of other examinations. The participants were positioned supine with their knees in full extension. Sequences included sagittal and coronal T2-weighted fat saturation fast spin echo (repetition time, 5000 ms; echo time, 80 ms; field-of-view, 16 cm; matrix, 288 × 288; and slice thickness, 3 mm with between-slice gaps of 1.0 mm). Evaluation of MRI scans was performed by two independent observers (DC and ES) using the whole-organ MRI score (WORMS) method^21^ in a blinded fashion, with no access to the participants’ clinical information. They recorded the presence of abnormalities, such as cartilage damage, BMLs, subchondral cysts, bone attrition, osteophytes, meniscal lesions, and synovitis in each subregion. In the WORMS system, the medial and lateral compartments of the tibia and femur are divided into three subregions (anterior, central, and posterior), and the tibia has one additional subregion, representing the area beneath the tibial spine. The patella is divided into medial and lateral subregions. Cartilage damage was scored in each of the 14 articular surfaces (excluding region S) from 0 (normal thickness and signal) to 6 (diffuse [≥ 75% of the region] full-thickness loss). BMLs were each scored as integers from 0 to 3, where 0 = normal; 1 = mild, < 25% of the region; 2 = moderate, 25–50% of the region; and 3 = severe, > 50% of the region. Subchondral cysts were graded in each region from 0 to 3 based on the extent of regional involvement, as for BMLs. Bone attrition was defined as flattening or depression of the articular surfaces and was graded from 0 to 3 based on the subjective degree of deviation from the normal contour: 0 = normal, 1 = mild, 2 = moderate, and 3 = severe. Osteophytes along 14 different margins of the knee, the anterior, central weight-bearing, and posterior margins of the femoral condyles and tibial plateaus, and the medial and lateral margins of the patella were graded from 0 to 7 using the following scale: 0 = none; 1 = equivocal; 2 = small; 3 = small-moderate; 4 = moderate; 5 = moderate-large; 6 = large; and 7 = very large. Meniscal lesions were graded separately from 0 to 4: 0 = intact; 1 = minor radial tear or parrot-beak tear; 2 = non-displaced tear or prior surgical repair; 3 = displaced tear or partial resection; and 4 = complete maceration/destruction or complete resection. Since joint effusion and synovial thickening cannot be distinguished from each other, synovitis was graded collectively from 0 to 3 in terms of the estimated maximal distention of the synovial cavity: 0 = normal; 1 = less than 33% of maximum potential distention; 2 = 33–66% of maximum potential distention; and 3 = more than 66% of maximum potential distention. For cartilage damage, BMLs, subchondral cysts, bone attrition, and osteophytes, we computed the number of subregions with damage as the number of subregions with a score of > 0. For meniscal lesions, we computed the maximum meniscal damage grade for the entire knee. The intra-rater and inter-rater reliabilities, expressed as ICC (1,1) and (2,1) of scoring MRI, were 0.929 and 0.921, respectively.

### Statistical analysis

The mean values of continuous variables (age, BMI, and KOOS) were compared using the Mann‒Whitney U test. The chi-square test was used to compare the prevalence of MRI lesions between the non-KOA and early KOA groups. To evaluate the relationship between knee symptoms and MRI lesions, we performed crude and adjusted logistic regression analyses, with the presence of knee symptoms as an independent variable, and age, BMI, and the total score of each MRI lesion as dependent variables. Furthermore, to evaluate the relationship between knee pain and MRI lesions, we performed multiple logistic regression analyses with the presence of knee pain as an independent variable, and age, BMI, and each MRI lesion as dependent variables. Data analysis was conducted using SPSS (version 24.0; IBM Corp., Armonk, NY, USA) in a cross-sectional manner. Statistical significance was set at *P* < 0.05.

## Results

### Participants’ demographics

**Figure 2:**
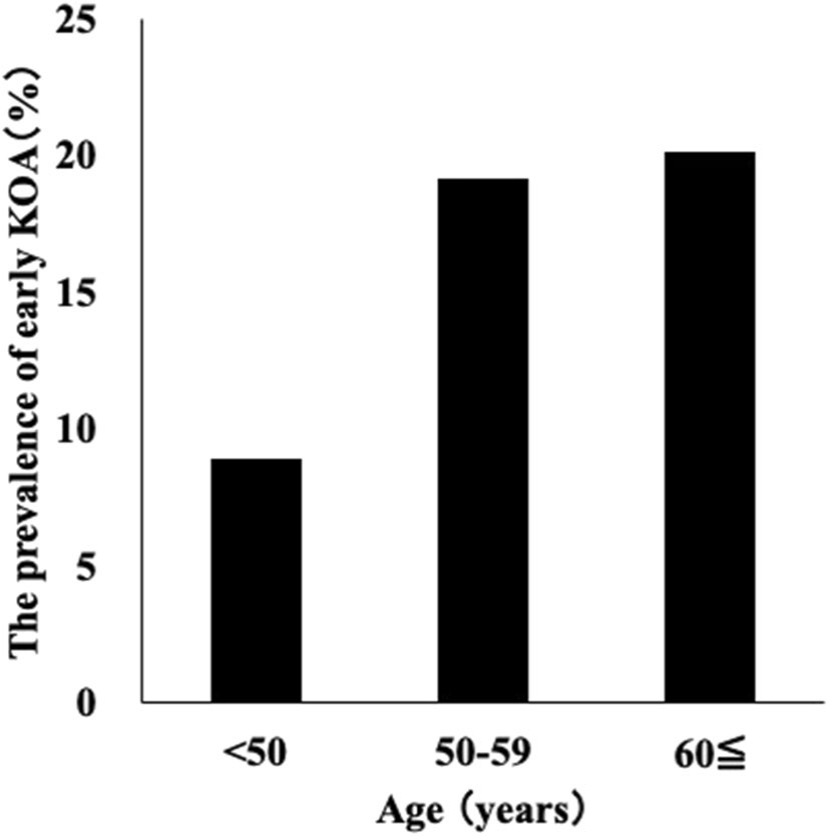
The prevalence of early knee osteoarthritis (KOA) classified by age.

The mean age of participants was 51.3 ± 11.7 years, mean BMI was 21.9 ± 3.1 kg/m^2^. Sixty-nine (19.2%) participants had symptomatic knees, and 54 (15.0%) met the diagnostic criteria for early KOA (Table 1). The prevalence of early KOA increased with age, exceeding 20% in participants aged 60 years and older (Fig. 2). The prevalences of cartilage damage, BMLs, subchondral cysts, bone attrition, osteophytes, meniscal lesions, and synovitis were 43.5%, 28.7%, 9.2%, 6.4%, 42.6%, 12.3%, and 25.9%, respectively. The severities of cartilage damage (*P* = 0.025), BMLs (*P* = 0.026), subchondral cysts (*P* = 0.048), bone attrition (*P* = 0.012), meniscal lesions (*P* < 0.001), and synovitis (*P* < 0.001) were significantly higher among participants with early KOA than among those without KOA (Tables 2, 3).

**Table 1:** The demographic data of study participants.

	Total sample (n = 359)	Non KOA (n = 305)	Early KOA (n = 54)	*p*-value
Age, years	51.3 ± 11.7	50.5 ± 11.8	55.9 ± 9.6	0.002
Body mass index, kg/m^2^	21.9 ± 3.1	21.8 ± 3.1	22.5 ± 3.2	0.101
**KOOS**				
Symptom	91.9 ± 10.8	94.8 ± 6.5	75.9 ± 15.4	< 0.001
Pain	93.4 ± 12.1	96.8 ± 6.9	74.0 ± 16.2	< 0.001
Physical function short form	95.1 ± 11.6	98.1 ± 5.4	77.8 ± 19.5	< 0.001
Quality of life	85.7 ± 18.7	90.8 ± 13.3	56.9 ± 18.7	< 0.001
Symptomatic knee, %	69 (19.2%)	15 (4.9%)	54 (100.0%)	< 0.001

Values are means ± standard deviations of age, body mass index, and KOOS. In regards to symptomatic knee, values are represented by prevalence (percentage of the whole population). Differences among the two groups were compared by Mann–Whitney U test and chi-square test. A *p*-value indicates the significance of difference between participants with and without knee symptoms. KOOS Knee injury and Outcome Score.

**Table 2:** The distribution of MRI lesions of study participants.

	Total sample (n = 359)	Non KOA (n = 305)	Early KOA (n = 54)	*p*-value
**No. of subregions with cartilage score > 0 across entire knee**				
0–1	312 (86.9)	271 (88.9)	41 (75.9)	0.025
2–4	46 (12.8)	33 (10.8)	13 (24.1)	
5 +	1 (0.3)	1 (0.3)	0 (0.0)	
**No. of subregions affected by any BML**				
0	256 (71.3)	226 (74.1)	30 (55.6)	0.026
1	75 (20.9)	58 (19.0)	17 (31.5)	
2	20 (5.6)	16 (5.2)	4 (7.4)	
3	4 (1.1	3 (1.0)	1 (1.9)	
4	3 (0.8)	1 (0.3)	2 (3.7)	
5 +	1 (0.3)	1 (0.3)	0 (0.0)	
**No. of subregions affected by any subchondral cyst**				
0	326 (90.8)	281 (92.1)	45 (83.3)	0.048
1	29 (8.1)	22 (7.2)	7 (13.0)	
2	4 (1.1)	2 (0.7)	2 (3.7)	

The values represent the number (percentage of the whole population) of MRI lesions of all participants, participants with early KOA, and those without KOA.

Differences among the two groups were compared by chi-square test.

A *p*-value indicates the significance of difference between participants with and without early KOA.

**Table 3:** The distribution of MRI lesions of study participants.

	Total sample (n = 359)	Non KOA (n = 305)	Early KOA (n = 54)	*p*-value
**No. of subregions affected by any attrition**				
0	336 (93.6)	290 (95.1)	46 (85.2)	0.012
1	23 (6.4)	15 (4.9)	8 (14.8)	
**No. of locations affected by any osteophyte**				
0–2	349 (97.2)	298 (97.7)	51 (94.4)	0.178
3 +	10 (2.8)	7 (2.3)	3 (5.6)	
**Maximum grade of meniscal lesion across all locations**				
0	315 (87.7)	276 (90.5)	39 (72.2)	< 0.001
1–2	33 (9.2)	24 (7.9)	9 (16.7)	
3 +	11 (3.1)	5 (1.6)	6 (11.1)	
**Synovitis**				
0–1	350 (97.5)	303 (99.3)	47 (87.0)	< 0.001
2–3	9 (2.5)	2 (0.7)	7 (13.0)	

The values represent the number (percentage of the whole population) of MRI lesions of all participants, participants with early KOA, and those without KOA.

Differences among the two groups were compared by chi-square test.

A *p*-value indicates the significance of difference between participants with and without early KOA.

### Association between early knee osteoarthritis and magnetic resonance imaging lesions

According to the logistic regression analysis, cartilage damage (odds ratio [OR] 1.463, 95% confidence interval [CI] 1.085, 1.974), BMLs (OR 1.499, 95% CI 1.098, 2.047), subchondral cysts (OR 2.201, 95% CI 1.112, 4.355), bone attrition (OR 3.362, 95% CI 1.350, 8.376), meniscal lesions (OR 1.836, 95% CI 1.332, 2.530), and synovitis (OR 2.779, 95% CI 1.736, 4.449) were significantly associated with the presence of early KOA. Furthermore, an adjusted regression model showed that meniscal lesions (OR 1.479, 95% CI 1.036, 2.110) and synovitis (OR 2.254, 95% CI 1.356, 3.747) were positively correlated with the presence of early KOA (Table 4).

**Table 4:** MRI lesion related to the presence of early knee osteoarthritis.

Independent variables	Non-adjusted			Adjusted		
	*p*-value	Odds ratio	95% CI	*p*-value	Odds ratio	95% CI
Cartilage	0.013	1.463	1.082–1.974	0.983	0.996	0.693–1.431
Bone marrow lesions	0.011	1.499	1.098–2.047	0.363	1.179	0.826–1.684
Subchondral cysts	0.024	2.201	1.112–4.355	0.155	1.711	0.816–3.591
Attrition	0.009	3.362	1.350–8.376	0.650	1.292	0.427–3.910
Osteophytes	0.092	1.297	0.958–1.757			
Meniscal lesions	< 0.001	1.836	1.332–2.530	0.031	1.479	1.036–2.110
Synovitis	< 0.001	2.779	1.736–4.449	0.002	2.254	1.356–3.747

Logistic regression analysis was performed on knees with Kellgren–Lawrence grade 0 or 1 in radiographs.

In non-adjusted model, dependent variables was the presence of early KOA; the independent variables were each MRI lesion.

In adjusted model, dependent variables was the presence of early KOA; the independent variables were age, BMI, MRI lesions except osteophytes.

### Association between knee pain and magnetic resonance imaging lesions

In terms of the association between MRI lesions and knee pain with certain motions, those with early KOA suffered from pain when going up or down stairs, bending the knee, or performing sitting or lying down motions (Table 5). Additionally, regression analysis showed that synovitis and meniscal lesions were positively associated with almost all KOOS pain items. On the other hand, cartilage was not significantly associated with any KOOS pain item, except for pain with going up or down stairs. BMLs tended to be associated with pain when straightening the knee (*P* = 0.067, OR 1.387, 95% CI 0.977, 1.968), bending the knee (*P* = 0.066, OR 1.340, 95% CI 0.981, 1.830), and walking on a flat surface (*P* = 0.039, OR 1.453, 95% CI 1.019, 2.073) (Table 6).

**Table 5:** The prevalence of each knee pains of study participants.

	Total sample (n = 359)	Non KOA (n = 305)	Early KOA (n = 54)	*p*-value
How often do you experience knee pain?	118 (32.9)	67 (22.0)	51 (94.4)	< 0.001
Twisting/pivoting on your knee	69 (19.2)	34 (11.1)	35 (64.8)	< 0.001
Straightening knee fully	43 (12.0)	16 (5.2)	27 (50.0)	< 0.001
Bending knee fully	68 (18.9)	27 (8.9)	41 (75.9)	< 0.001
Walking on flat surface	38 (10.6)	16 (5.2)	22 (40.7)	< 0.001
Going up or down stairs	87 (24.2)	44 (14.4)	43 (79.6)	< 0.001
At night while in bed	25 (7.0)	11 (3.6)	14 (25.9)	< 0.001
Sitting or lying	73 (20.3)	34 (11.1)	39 (72.2)	< 0.001
Standing upright	38 (10.6)	18 (5.9)	20 (37.0)	< 0.001

The values represent the prevalence of each knee pains of all participants, participants with early KOA, and those without KOA.

Data are based on the number of participants (percentage of the whole population) for each knee pains.

Differences among the two groups were compared by chi-square test.

A *p*-value indicates the significance of difference between participants with and without early KOA.

**Table 6:** The association between MRI lesions and knee pains with certain activities.

	Cartilage	Bone marrow lesions	Subchondral cysts	Attrition	Ostcephytes	Meniscal lesions	Synovitis
How often do you experience knee pain?	1.11 (0.85–1.45)	1.17 (0.88–1.56)	1.05 (0.55–2.00)	**2.51 (1.05–5.99)**	1.07 (0.82–1.39)	**1.73 (1.22–2.44)**	**2.04 (1.34–3.11)**
Twisting/pivoting on your knee	1.09 (0.80–1.48)	1.26 (0.92–1.72	1.17 (0.57–2.41)	**3.24 (1.34–7.84)**	1.32 (0.99–1.76)	**1.51 (1.11–2.06)**	**1.70 (1.09–2.65)**
Straightening knee fully	0.94 (0.64–1.38)	1.39 (0.98–1.97)	0.97 (0.39–2.39)	1.43 (0.45–4.49)	1.16 (0.82–1.64)	**1.41 (1.01–1.98)**	**1.82 (1.10–2.98)**
Bending knee fully	1.16 (0.86–1.57)	1.34 (0.98–1.83)	1.35 (0.67–2.72)	2.10 (0.84–5.26)	1.27 (0.95–1.70)	**1.59 (1.16–2.17)**	**1.65 (1.06–2.58)**
Walking on flat surface	0.97 (0.65–1.45)	**1.45 (1.02–2.07)**	1.10 (0.45–2.72)	2.22 (0.76–6.51)	1.11 (0.77–1.60)	1.23 (0.84–1.79)	**2.01 (1.20–3.35)**
Going up or down stairs	**1.35 (1.02–1.79)**	1.26 (0.93–1.70)	1.72 (0.90–3.29)	**3.51 (1.46–8.43)**	**1.49 (1.13–1.96)**	**1.78 (1.27–2.48)**	**2.08 (1.35–3.20)**
At night while in bed	1.18 (0.76–1.83)	1.33 (0.85–2.07)	0.64 (0.16–2.52)	2.11 (0.56–7.91)	**1.72 (1.17–2.51)**	**1.59 (1.10–2.29)**	1.51 (0.81–2.80)
Sitting or lying	0.97 (0.72–1.32)	1.20 (0.88–1.63)	1.13 (0.55–2.31)	1.71 (0.68–4.34)	1.11 (0.83–1.50)	1.15 (0.83–1.59)	**1.97 (1.26–3.07)**
Standing upright	0.79 (0.51–1.24)	1.16 (0.78–1.72)	1.11 (0.45–2.74)	1.68 (0.53–5.32)	1.28 (0.90–1.82)	**1.51 (1.08–2.11)**	**1.86 (1.11–3.12)**

Logistic regression analysis was performed on knees with Kellgren–Lawrence grade 0 or 1 in radiographs. Dependent variables was the presence of each knee pains; the independent variables were age, BMI, and each MRI lesion. Bold denotes statistical significance at *P* < 0.05.

## Discussion

The most important findings of the present study were that MRI findings in early KOA diagnosed by the newly established criteria were proven, and imaging evidence of various types of knee pain was demonstrated. Although MRI evaluation showed that cartilage damage (44%) was the most common finding in women without radiographic knee abnormalities, there was no significant association with the presence of early KOA. The prevalence of BMLs (29%), subchondral cysts (9%), bone attrition (6%), meniscal lesions (12%), and synovitis (26%) were higher in patients with early KOA than in those without. Meniscal lesions and synovitis were positively associated with the presence of early KOA and key factors for various types of knee pain. These results suggest that meniscal lesions and synovitis have the greatest impact on the symptoms of patients with early KOA, thus proving our hypothesis; these should be detected and appropriate intervention should be applied to prevent disease progression.

Few studies have investigated the prevalence of abnormalities detected by MRI, specifically in a population without radiographic KOA. Guermazi et al. reported that osteophytes were the most common abnormality (74%), followed by cartilage damage (69%), BMLs (52%), synovitis (37%), and bone attrition (32%) in participants without radiographic evidence of KOA^22^. Javaid et al. found that osteophytes (99%), cartilage damage (70%), and BMLs (59%) were common in participants without knee symptoms and radiographic KOA^23^. In the current study, the same tendency was observed, that is, many participants had cartilage damage, osteophytes, and BMLs. However, the prevalence of each abnormality was lower than that reported in previous studies. This may be because the participants in our study were younger, had a lower BMI, and might have a lower risk of KOA than those in previous studies.

Many previous studies have reported an association between knee effusion and knee symptoms. Torres et al. noted that synovitis or effusion detected on MRI correlated best with knee pain measured on a visual analog scale^24^. Chiba et al. investigated suprapatellar effusion using quantitative ultrasonography and concluded that knee effusion was associated with symptoms as evaluated by KOOS^13^. Moreover, several studies have shown that knee effusion is associated with the KOA disease stage and progression^14,25,26^. Although there have been few studies of effusion in early KOA, Harkey et al. suggested that effusion precedes the onset of accelerated KOA and may be a prognostic biomarker^27^. In the present study, synovitis was the most strongly associated with knee symptoms and was considered to be the most important clinical finding indicating the need for intervention, which is in agreement with findings of previous reports.

Meniscal lesions and BMLs have also been associated with knee symptoms and disease progression in previous studies^28–31^. In addition, there have been reports that meniscal lesions, BMLs, and synovitis are associated with each other^32–34^. However, van Oudenaarde et al. suggested that the discriminative power of single MRI features is insufficient to be useful as a predictor of KOA^35^. In our study, although meniscal lesions, together with cartilage damage, BMLs, subchondral cysts, bone attrition, and synovitis, were significantly associated with the presence of early KOA, the adjusted regression model showed that this association was attenuated after adjustment for age and BMI. In the early KOA population, synovitis contributes more to knee symptoms than meniscal lesions.

BMLs reflect bony damage, which is associated with knee symptoms in patients with KOA and bone metabolism, such as bone absorption. In recent studies, the presence of BMLs has been associated with knee pain and predicted cartilage loss in patients with established KOA^36–38^. Antiresorptive drugs (e.g., bisphosphonate) were found to reduce the size of BMLs and the risk of total knee arthroplasty in patients with KOA^39,40^. In early KOA, lower bone mineral density and higher levels of some bone markers were associated with the presence of BMLs^15^. In the present study, BMLs tended to be associated with pain in relatively loaded motions, such as bending and straightening the knees and walking. BMLs may be induced by bone fragility, resulting in early subchondral changes, such as microcracking before radiologic osteoarthritic findings become definitive and cause pain during loading.

In many previous studies, although cartilage damage was associated with bone marrow lesions^41,42^, meniscal damage^43^, and synovitis or effusion^44^, cartilage damage was not always associated with the severity of knee pain^22^. Since articular cartilage is not innervated, the association between cartilage and pain severity may be due to other structural abnormalities associated with KOA^45^. In this study, cartilage damage was the most common finding, but it was not significantly associated with pain. This result suggested that minor cartilage damage detected by MRI was not associated with knee symptoms in accordance with the established KOA.

This study had several limitations. First, all participants in this study were women. Although the prevalence of KOA is higher in women than in men, men may be more likely to experience knee trauma, which may affect the prevalence of cartilage damage, BMLs, and meniscal lesions. Second, laboratory data, including inflammation and cartilage/bone metabolic markers, were not evaluated. Serum hyaluronic acid, a marker of synovitis, has been assessed in many previous studies^14,46^. Third, the data of synovitis were obtained using non-contrast images, which are less sensitive in distinguishing effusion from underlying synovial tissue than contrast images. Because of the possible complications and increased cost, we did not use contrast-enhanced MRI. Fourth, the findings of this study were based on cross-sectional data; therefore, future longitudinal studies are needed to investigate whether patients with knee effusion are likely to develop KOA. In addition, because of some findings, such as repeated remission and exacerbation of BMLs, caution may be required when interpreting the results of this study.

## Conclusions

Structural abnormalities detected by MRI, such as cartilage damage, osteophytes, and BMLs, were commonly present in the knees of middle-aged Japanese women with no radiographic evidence of KOA. Among MRI findings, synovitis had the most marked effect on knee pain and might be a therapeutic target in patients with early KOA.

## Data Availability

The data to reproduce the analyses are available at https://drive.google.com/drive/folders/1Ch7_IH8cDUdYiitqIABBsAfHosZbUsMm?usp=sharing.
